# Vision screening in children: a retrospective study of social and demographic factors with regards to visual outcomes

**DOI:** 10.1136/bjophthalmol-2015-307206

**Published:** 2015-11-23

**Authors:** U O'Colmain, L Low, C Gilmour, C J MacEwen

**Affiliations:** 1 Department of Ophthalmology, Ninewells Hospital and Medical School, Dundee, Scotland, UK; 2 Academic Unit of Ophthalmology, University of Birmingham, Birmingham, UK

**Keywords:** Epidemiology, Child health (paediatrics), Optics and Refraction, Vision

## Abstract

**Background:**

Amblyopia and its risk factors have been demonstrated to be more common among children from low socioeconomic backgrounds. We sought to investigate this association in a region with orthoptic-delivered screening and whole population coverage, and to also examine the association of the Health Plan Indicator (HPI) with screening outcome.

**Methods:**

Screening examination outcomes, postcodes and HPIs were extracted from the community child health database for every child who underwent preschool vision screening between March 2010 and February 2011 Tayside. We obtained the Scottish Index of Multiple Deprivation score for every child as a measure of area-based deprivation. We assessed the vulnerability/needs of the individual family through the HPI—‘Core’ (children and families receiving universal health visiting service), ‘Additional’ (receiving additional health/social support) and ‘Intensive’ (receiving high levels of support). The outcomes from follow-up examinations for those who failed screening were extracted from the orthoptic department database.

**Results:**

4365 children were screened during the year 2010–2011 of whom 523 (11.9%) failed. The odds of children from the least deprived socioeconomic group passing the visual screening test was 1.4 times higher than those from the most deprived socioeconomic group (OR 1.4, 95% CI 1.07 to 1.89, p=0.01). The odds of a child from a family assigned as ‘Intensive’ failing the preschool visual screening test was three times greater than the odds of a child from a family assigned as ‘Core’ (OR 3.59, 95% CI 1.6 to 7.8, p=0.001).

**Conclusions:**

We found that children from the most deprived backgrounds and those from unstable homes were more likely to fail preschool vision screening.

## Introduction

The National Screening Committee and the Hall (Four) Report recommend orthoptic-led vision screening for children between the ages of 4 and 5 years old.^1^ ^2^ Testing children at this age by trained practitioners has been identified as the most effective and cost-efficient way to capture children at risk of amblyopia and associated visual problems.^2^

Amblyopia is a preventable cause of visual disability and may have an impact on a child's education and behaviour:^3^ cosmetically poor strabismus is independently acknowledged to be unacceptable in most societies due to its social and psychological impact.^4^ Amblyopia and strabismus may have an impact on the future eye health and social opportunities of those affected.^3^ ^5^

A number of studies have described an association between lower socioeconomic status and the incidence of paediatric eye conditions including refractive error, strabismus and amblyopia. These studies have used self-reported and objective measures of socioeconomic status.^6–8^ Conclusions have also been drawn about inequity of access to healthcare with children from more deprived backgrounds being less likely to access an eye health professional.^6^ ^9^ ^10^

In the UK, all newborn infants have a postnatal screening examination of red reflex and for major eye abnormalities; the next examination is provided at 6–8 weeks of age, usually undertaken by the General Practitioner (GP) who examines the baby for normal visual behaviour, the presence of a red reflex and eye abnormalities. The next and final vision screening examination for children is recommended to take place at the age of 4–5 years.

The aim of this study is to report the outcomes of a cohort of children screened by the Tayside Preschool Vision Screening (PSVS) programme and to compare their socioeconomic and home backgrounds with the outcomes of vision screening.

## Methods

The details of every child living in our region are stored from birth on the community child health database; the database includes their demographic details, Health Plan Indicator (HPI), address/postcode and the result of their PSVS test. The child health database also includes information on each child's public health record. In Tayside, a region in the east of Scotland, there is a long established orthoptic-delivered vision screening programme for children between 4 and 5 years of age—the PSVS programme.

### Definitions

#### Socioeconomic deprivation

A. Scottish Index of Multiple Deprivation

We used the Scottish Index of Multiple Deprivation (SIMD) 2012 score as a measure of area-based deprivation. We used the residential postcodes to assign every child an individual SIMD 2012 score. The higher the SIMD score, the more deprived the area. We used the SIMD score to group the data into quintiles: 0–20% most deprived, 20–40%, 40–60%, 60–80%, 80–100% least deprived. To examine the link between extreme deprivation and vision screening results, we subdivided the SIMD groups into 0–20% most deprived areas and 20–100% least deprived areas.

B. Health Plan Indicator

The HPI is based on a comprehensive assessment of the needs of the child and family's circumstances and situations. It is a code applied to every child in the UK before or around the time of birth by their assigned health visitor (HV). Specifically, it is calculated based on an assessment of the stability and safety of the child's home and family; the function of the indicator is to ensure the provision of the appropriate level of support that will be necessary for the child. There are three HPI codes: Core (C), Additional (A) and Intensive (I). A child from a normal, stable home about which there are no concerns would be assigned ‘Core’ and would receive routine HV and GP input; a child from a less stable home, for example a single young mother with poor family support, could be assigned ‘Additional’—she and her child would receive additional input from the HV, social worker and GP; a child from an unstable, chaotic home, for example with substance abuse problems, could be assigned ‘Intensive’—these children and families receive high levels of contact and input from health and social services. The possible impact of the stability and security of a child's home environment has not previously been reported, and the HPI is the only formally applied measure of this factor which is widely used and recognised.

Demographics, postcode and HPI data were extracted from the community child health database for every child who underwent preschool vision screening between March 2010 and February 2011. Each child's SIMD was calculated using a tool provided on the Scottish government website using their home postcode. The outcomes of the screening examination and referral visit were extracted from the orthoptic PSVS database and long-term follow-up visual and refractive data were extracted from the clinical notes.

The HPI and SIMD may change if a family's situation changes or if they move house, and while the database is maintained ‘live’ and up to date, we used the codes/scores on the database at the time of the child's birth.

Preschool vision screening in Tayside is a single programme which covers the whole population; children aged between 4 and 5 years are screened. It is orthoptist-delivered—achieved in the majority of cases by orthoptists visiting childcare nurseries/preschool classes. If a child does not attend nursery/preschool they are invited to attend a clinic at one of the community health centres or in the hospital, whichever is more local to them. If a child fails screening, they are referred to a clinic either at the community health centre or the hospital. At the referral clinic visit their visual acuity and orthoptic examination is rechecked, they undergo cycloplegic refraction and ocular examination by a hospital optometrist or ophthalmologist. Follow-up is arranged in the same community/hospital clinics if required.

The vision standard to pass screening is vision of greater than 0.2 LogMAR (Keeler test), or greater than 0.1 LogMAR with crowded Kay pictures if ‘letter’ testing is not achieved. A cover test, ocular motility, convergence, prism reflex test and stereotest are also carried out. These tests give additional information to the orthoptist about binocular function and they allow detection of ocular motility disorders and other abnormalities such as anisocoria. If a child has already been referred to the hospital eye service prior to their PSVS, they are recorded as ‘under review’ which counts as a fail for epidemiological purposes if the reason they are under review relates to any visual/refractive/strabismic diagnosis.

We examined the details of every child who failed the PSVS in the screening year 2010–2011 (children born 2006–2007) (n=523) and, as a control group, examined the details of 647 randomly chosen children who passed their PSVS the same year.

### Statistical analysis

We analysed the data using the SPSS statistical package (IBM SPSS Statistics for Windows, V.19.0, IBM Corp, Armonk, New York, USA). We used the χ^2^ test to calculate the association between visual screening outcome and socioeconomic background based on SIMD index and HPI index, respectively. We used one-way analysis of variance to assess the association between the mean LogMar visual acuities and socioeconomic background based on the SIMD index and HPI index, respectively.

## Results

### Tayside preschool vision screening programme

Four thousand three hundred and sixty-five children were screened during the year 2010–2011; 523 (11.9%) failed and were referred for repeat orthoptic examination, cycloplegic refraction and ocular examination. The results of the screening examination—visual acuities and orthoptic examination—were complete for 493/523 (94.3%) children.

**Figure 1:**
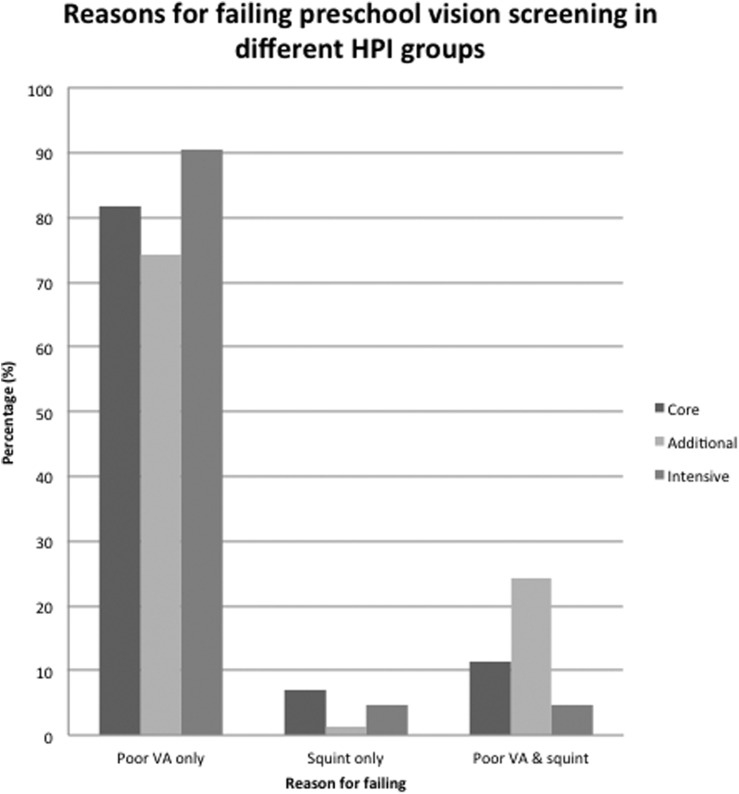
This graph shows the reasons for failing preschool vision screening according to the Health Plan Indicator (HPI) categories.

**Figure 2:**
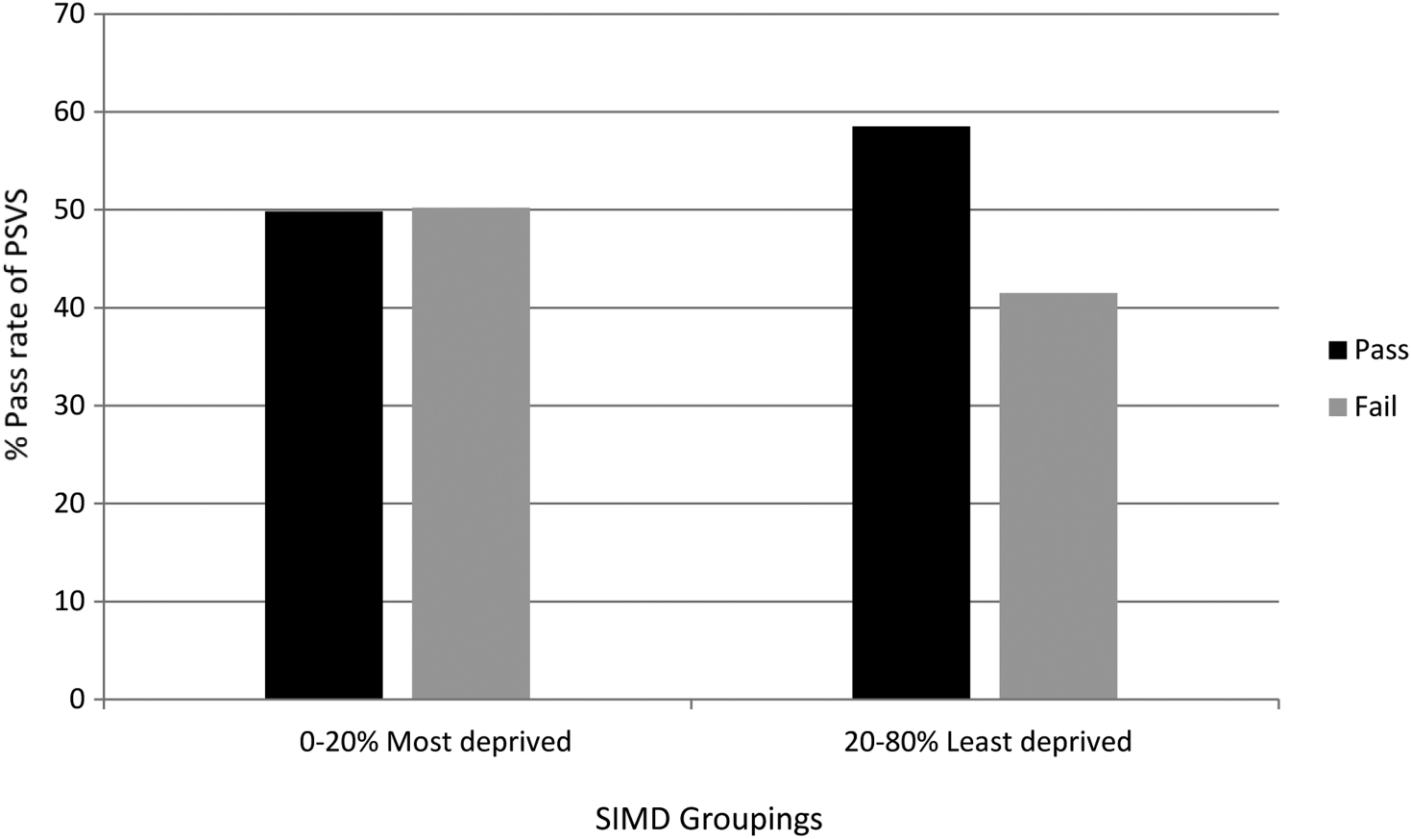
This graph shows the percentage of children passing or failing the vision screening test divided by the Scottish Index of Multiple Deprivation (SIMD) group. The y axis represents the percentage of children passing their PSVS, while the x axis represents the SIMD groups from 0–20% most deprived to 20–100% least deprived socioeconomic groups.

**Figure 3:**
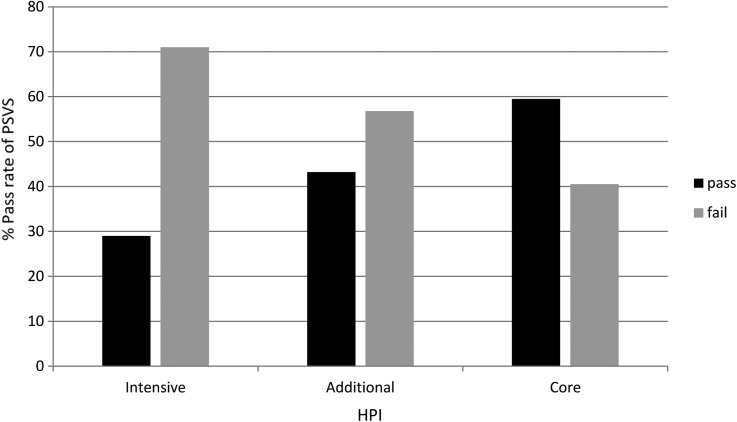
This graph shows the percentage of children passing or failing the vision screening test divided by the Health Plan Indicator (HPI) group.

1. The reasons for failing screening.
  The most common reason for a child to fail PSVS was reduced visual acuity 399/493 (80.9%); 65/493 (13.2%) failed due to reduced visual acuity (VA) and strabismus; 29/493 (5.8%) had strabismus alone (with normal/pass level VA) (Figure 1). The mean level of vision in the better eye of those with bilaterally reduced vision was 0.36 LogMAR and the mean level of vision of those with uniocular reduced vision was 0.44 LogMAR in the failing eye.
  - In children who failed screening due to reduced vision only, there was a weak correlation between poor vision and socioeconomic deprivation according to SIMD rank. The correlation between vision in the better eye and SIMD score was r=−0.005, p value of 0.91. The correlation between vision in the worse eye and SIMD score was r=0.079, p value 0.097.
2. The odds of passing PSVS test according to SIMD.
  The odds of children from the 20% to 100% least deprived socioeconomic group passing the visual screening test was 1.4 times higher than those from the 0% to 20% most deprived socioeconomic group (OR 1.4, 95% CI 1.07 to 1.89, p=0.017). This result was independent of the child's HPI (figure 2).
3. The odds of passing PSVS test according to HPI
  The odds of a child from a family assigned as ‘Intensive’ failing the preschool visual screening test was three times greater than the odds of a child from a family assigned as ‘Core’ (OR 3.59, 95% CI 1.6 to 7.8, p=0.001). This result was independent of the child's SIMD (figure 3).

### Secondary hospital eye service follow-up visit

- Failure to attend
  - The results of the secondary hospital referral (repeat orthoptic examination, refraction and examination) were unavailable on 93/523 (17.7%) children either because they did not attend their follow-up appointment or no data were available from that visit. This left 430 children on whom there were screening and follow-up information.
  - Among children who never attended their referral appointment after failing PSVS there was a trend towards a higher proportion with HPIs of ‘Intensive’. This was not statistically significant due to the small number of cases.
- Outcome of the secondary hospital eye service follow-up visit
  Two hundred and fifty-four (59.1%) children were prescribed glasses at their referral visit. The remaining 176 (40.9%) were not prescribed glasses, of whom 57 were discharged because their repeat vision test was within ‘pass’ standards. One hundred and nineteen children were kept under review.

## Discussion

Previous studies on the impact of socioeconomic factors on vision and visual defects have repeatedly found an association between lower socioeconomic background and poor vision.^6–9^ These studies used various indices of deprivation, for example, *the Noble*, and parent-reported data on occupational class. In Scotland the HPI is a universally applied index which is objectively applied by a HV who is familiar with the family. To our knowledge, this is the first time that a personalised indicator of social background such as HPI has been used to illustrate the differences in pass rates of children’s vision screening.

Our data demonstrate a significant disparity between the most deprived 20% of children and the least deprived 80%, with children from the most deprived geographical areas significantly more likely to fail screening. We also found that independent of geographical area, a child's home situation was correlated with failure—with ‘Intensive’ support children significantly more likely to fail their vision screening than ‘Core’ support children.

The preschool screening programme is designed to pick up amblyopia and its risk factors, such as strabismus and refractive error, at a stage where detection and treatment rates are effective. It has been recognised that children from deprived backgrounds have higher rates of refractive error and amblyopia.^6^ Our study supports this claim. In addition, we have investigated the aspect of home stability as indicated by the HPI. This is the first time this measure of family circumstances has been examined as an associated factor with vision.

Although there is not enough biological evidence to explain why children from the ‘Intensive’ group were more likely to fail the vision screening test, several pieces of evidence from epidemiological studies suggest that inadequate prenatal/antenatal care, more commonly in socioeconomically deprived families, may contribute to poorer visual outcomes in children.^9^ ^10^ First, maternal smoking during pregnancy, which is more prevalent among the lower social class, single mothers and those on benefits,^11^ has been associated with strabismus.^12^ ^13^ Second, the increased incidence of vision problems among children of women who consume drugs and alcohol during pregnancy may be relevant^14^ ^15^ as there is an increased antenatal drug and alcohol usage in deprived areas,^16^ and these children would be assigned ‘Intensive’ HPIs.

Our findings have implications for future planning of resource allocation and the provision of healthcare. We previously reported unequal distribution of optometry practices within our region—there were fewer practices located in more deprived areas with poor geographical access compared with affluent areas with good geographical access.^17^ Taken in context of the trend towards a higher rate of non-attendance at referral appointments among children from deprived backgrounds, these children are at an increased disadvantage—with poor local access to primary eye healthcare tied on with parental failure to bring them to appointments. In Scotland optometrists work under an enhanced General Ophthalmic Services contract whereby they provide primary eye healthcare services; it is therefore desirable that optometry premises are accessible to all population groups, in the same way that GP practices are.^17^

The strengths of our study are that our PSVS is orthoptist-delivered, and has whole population coverage. We have a high rate of complete data from the screening test and the follow-up visit. Additionally we report the area-based deprivation status and the HPI correlations with failing the PSVS, independent of each other, which we believe make our data more robust. The false-positive and non-attendance rates are acceptable for a screening programme. All patients were seen under the auspices of the National Health Service (NHS) optometry service and only clinically significant refractive errors were prescribed glasses. All children underwent cycloplegic testing. The population of Tayside is homogeneous, white/Scottish population so we do not believe that there is an effect from geographical concentration of ethnic groups. As the number of live births in NHS Tayside between 1 April 2006 and 31 March 2007 were 3665,^18^ we are confident that we achieved high population coverage of preschool vision screening. In addition, all eligible children are screened within a 12-month period, and they are within 12 months of age of each other. If the child fails to attend screening, they are recalled later in the year. We are therefore confident that the results of our study are not just a mere effect of the delayed screening in socioeconomically deprived populations. One limitation of the study is the retrospective nature of the data collection.

## Conclusion

We report the outcomes of a large cohort from an orthoptic-delivered preschool vision screening service encompassing all children in our region. We found a significant disparity in passing vision screening between the most deprived 20% of children and the least deprived 80%, with the most deprived more likely to fail.

We also found that children from more chaotic home backgrounds, conferring a HPI rating of ‘Intensive’ on the child, are more likely to fail screening.

These results are important for planning resource management in public health and screening support.
